# A Theoretical Framework for Estimating the Rate of Return to a Pharmacy Education Anywhere

**DOI:** 10.3390/pharmacy8030162

**Published:** 2020-09-03

**Authors:** Manuel J. Carvajal, Ioana Popovici

**Affiliations:** Department of Sociobehavioral and Administrative Pharmacy, College of Pharmacy, Nova Southeastern University, Fort Lauderdale, FL 33328, USA

**Keywords:** education, human-capital investment, pharmacist workforce, rate of return, wages and salaries

## Abstract

Undertaking a pharmacy education is an investment in human capital. Candidates trade off present versus future costs and benefits. They make this investment with the expectation of earning enough income throughout their worklives to make their undertaking financially worthwhile. Whether or not this occurs is determined by the rate of return. The aim of the current study was to construct a theoretical model to estimate the rate of return to a pharmacy education investment. Specifications for model assumptions, inputs, and outputs are discussed. The outputs are the rates of return, the inputs are the costs and benefits of a pharmacy education, and the assumptions illustrate the circumstances of the individual or group for whom the model is built. The rate of return is the annual percentage that equates the streams of benefits and costs over the investment span. The higher the value of the rate of return to a pharmacy education is, the more profitable is the investment. This theoretical model may be used to estimate the financial viability of pharmacy and compare it to the viability of other professions or to the viability of pharmacy among various locations.

## 1. Introduction

When candidates being interviewed for admission into pharmacy school are asked why they want to become pharmacists, they frequently answer that they want to help people, that they were inspired to do so by a sick-relative event, or some other altruistic response. Rarely do members of admission panels hear a pecuniary-based answer such as “I want to make money,” “jobs are good and plentiful,” or “because wages and salaries are competitive.” There seems to be an unwritten mandate to conceal the motive for financial gain, as if it were pedestrian or uncouth to admit that potential students weigh the costs and benefits of a pharmacy education and subsequent career in medication management vis-à-vis studying other disciplines, or not studying at all, within the context of their aptitudes, interests, preferences, dreams, and constraints. Concealing the financial motive when applying for admission, or at least not expressing it overtly, however, neither removes its existence nor diminishes its importance in the students’ decision-making process.

Undertaking a pharmacy education is an investment in human capital. (The process in this article is applied to the pharmacy profession, but it also is applicable to other types of education.) It is an investment because candidates must make decisions based on initial and later costs as well as on tradeoffs between present and future benefits. Candidates make a commitment to invest in themselves time and money to expand the endowments and skills with which they were born, and which they developed and refined during the years prior to attending pharmacy school. They make this commitment driven by the expectation of becoming more productive workers and receiving enough income throughout their worklives to make their investment financially worthwhile [1]. Whether or not this actually occurs is determined by the rate of return [2].

The purpose of this paper was to develop a model to estimate the expected rate of return to a pharmacy education investment anywhere in the world. This is the annual percentage that equates the present value of lifetime benefits from practicing pharmacy to the cost of being trained as a pharmacist and receiving a practicing license. Several studies have dealt with related issues over the last 25 years. In the USA, Mott and Kreling [3] examined the relationship between the supply of pharmacists and the internal rate of return; Cain et al. [4] focused on tuition, student debt, job potential, and pharmacists’ salaries; and Chisholm-Burns et al. [5] established that investing in a pharmacy education yields a positive rate of return. More recently, Carvajal and Popovici [6] estimated this rate to be 4.89% per annum at 2019 prices, which was equivalent to 6.44% per annum when inflation was included. Outside of pharmacy, the rate of return has been used to study issues related to the education of physicians [7], dentists [8,9], and nurses [10,11,12].

The methodology developed here is intended as a frame of reference that may be used to compare the financial viability of pharmacy to the viability of other educational and training pursuits. It relies solely on tangible costs and benefits that may be measured objectively, and does not consider intangible factors such as job or career satisfaction/dissatisfaction after graduation, preferences for pharmacy school or academic curriculum, or passions and challenges experienced during schooling or subsequently in professional life. Any of these factors is likely to be interpreted radically different by different individuals, so there would be no common denominator. Instead, the analysis is circumscribed to whatever may be measured in dollars, euros, or whatever other currency of choice, hereafter called currency units (CU). The framework has been applied successfully to estimate the rate of return to a pharmacy education investment in the USA [6].

## 2. Methods

Building a model requires specification of outputs, inputs, mechanisms, and assumptions. Here the outputs are the rates of return; the inputs are the costs and benefits related to the pharmacy education investment; the mechanisms are the ways in which costs and benefits interact with one another, which are further explained in the Results section; and the assumptions capture the circumstances pertaining to the individual or group for whom the model is built. The assumptions determine the choice and levels of costs and benefits to be used when the model is applied. The value and accuracy of the estimates depend on the choice of assumptions. Different inputs and/or input levels, due to different sets of assumptions, are likely to yield different rates of return. One set of assumptions is not necessarily better or worse than any other; each simply depicts a specific situation or batch of circumstances that conforms to one or another individual or group.

At the extremes, models may be simplistic or excessively complex. On the one hand, a simplistic model has the advantage of being easy to manipulate, but it generally is not able to capture the complexities of a real-life situation; in other words, it might not be useful to make a prediction. On the other hand, an excessively complex model is usually unmanageable (i.e., also useless). A satisfactory compromise between both extremes must be found at each step of the way.

### 2.1. Step 1. Model Output: Choice of Nominal or Real Rate of Return

The nominal rate of return includes the effect of changing prices over time, that is, the rate of inflation. Inflation normally is tracked by changes in a consumer price index, which measures periodic variations in the price level of a typical basket of goods and services purchased by households in a country or region. The estimation of a nominal rate of return requires that costs and benefits be expressed with changing prices through time. Thus, if a certain cost or benefit is worth 100 CU in Year 1 (i.e., base year), and the annual rate of inflation is 3%, the same cost or benefit would be equivalent to 103 CU in Year 2, 106.09 CU in Year 3, and so on.

The real rate of return adjusts for inflation, so it portrays a more realistic view of the purchasing power of the return to the investment. It is estimated using constant prices, at the base year, usually fixed at the beginning of the investment period. Calculating a real rate of return eliminates the tedious procedure of converting the value of expected costs and benefits at any given year into past- or future-value equivalents, or vice-versa, across the investment span. A value expressed for any year has the same purchasing power as the value in the base year.

The nominal rate of return poses the advantage over the real rate of return that the assumed rate of inflation may change over different years or groups of years; for example, expected benefits may be subject to a 3% inflation rate for the first five years after graduation, 6% for the next five years, etc.; the average inflation rate over the investment horizon would be equivalent to the compounded rate throughout the years. In the end, the real rate of return is equal to the nominal rate of return minus the inflation rate, so if the nominal rate of return is 10% and the inflation rate is 3%, the real rate of return would be 7% per year.

The annual inflation rate(s) used along the span of the investment may be actual or estimated, depending on the person or group for whom the model is built. For example, if the rate of return is being calculated for a pharmacist near retirement, most of the annual inflation rates entered into the model would be historically recorded values, while the data used for a potential student would entail a projection of annual inflation rates based on values recorded in previous years and/or anticipated economic conditions.

### 2.2. Step 2. Model Assumptions: Personal Characteristics

The personal characteristics of the individual or group undertaking the pharmacy education investment are important because they affect the types and levels of expected costs incurred and/or benefits received. One such characteristic is the age at which the person commences pharmacy school, which conditions the age at which he/she is graduated and begins working as a pharmacist. Another characteristic is the expected retirement age. For an older pharmacist, retirement age may be definitive (i.e., already decided), but for a younger student retirement age is a fuzzier prospect that lies decades away. The current retirement age for social insurance purposes may not be appropriate for the calculation, since rising life expectancy over time may increase the age at which workers begin receiving retirement benefits; thus, changes in the pertinent retirement age in the model, along with their justification, seem to be in order. In any event, the difference between graduation (plus perhaps a buffer period leading to the first job as a pharmacist) and retirement constitutes the pharmacist’s potential worklife span. Other things equal, the longer the worklife span is (i.e., because of younger graduation age and/or older retirement age), the higher the expected aggregate benefits from the investment are likely to be.

Another important variable is the length of the pharmacy training program. A longer curriculum carries with it higher direct and indirect costs. Also important is whether or not graduates must pass a licensing exam by an accredited body that may demand months of preparation or waiting. Furthermore, some countries or regions may require post-graduation internship service at below-market wages and salaries, which would decrease the aggregate stream of benefits. Still another consideration is the nature of prerequisites for entering pharmacy school. In some countries or regions students are eligible to enter pharmacy school with a secondary education degree, while in others a baccalaureate degree and/or specific courses may be required, thereby lengthening the training period and reducing the worklife span. Gender, willingness and ability to work, and job-related preferences also influence long-term earnings, which in turn affect the rate of return.

### 2.3. Step 3. Model Inputs: Costs and Benefits of the Pharmacy Education Investment

#### 2.3.1. Costs

In countries or regions where tertiary education is not free, or for students attending private universities, tuition is the largest source of direct cost. To calculate total tuition expenditures, either a constant annual tuition amount is multiplied by the number of years of the training program or, if there are tuition value variations due to inflation or other reasons, the actual or projected numbers for each year are recorded. A representative annual fee for books, materials, transportation to and from the university, and other school-related expenses should be added. General living expenses such as housing, meals, entertainment, etc. should not be included because they would be incurred if the person did not attend pharmacy school.

Oftentimes students need to borrow money to pay for tuition and other expenses while they attend pharmacy school. The amount they borrow is not considered an investment cost (i.e., that would be double counting), but the interest they pay every year, while all or part of the loan is outstanding, is considered a cost of financing the education investment. The total cost of financing depends on the amount borrowed, the interest rate of the loan, and how fast after graduation the loan is paid. A word of caution is warranted here: There is a difference between the nominal rate of interest and the real rate of interest (i.e., adjusted for inflation) that should be taken into account when estimating nominal and real financial costs.

Another consideration is the opportunity cost, which is the cost of the next best alternative of a person’s time. This is an imputed value, not an out-of-pocket expense. If the individual had not gone to pharmacy school and become a pharmacist, he/she might have worked in another job or occupation for which he/she was qualified prior to undertaking the pharmacy education investment; therefore, the salary that he/she would have earned in the other job or occupation is the pharmacist’s opportunity cost. For example, some colleges of pharmacy require that the student earn a baccalaureate degree (i.e., chemistry, biology, etc.) prior to entering pharmacy school. The student’s, and subsequently pharmacist’s, opportunity cost in this case would be the income that he/she might have earned as a chemist, biologist, etc. during the pharmacy school years as well as the worklife span. Obviously, better skills and qualifications prior to becoming a pharmacist would increase the opportunity cost, which in turn would reduce the investment rate of return.

Also important is to allow an annual percentage increment in the opportunity cost to account for productivity gains experienced in the alternative job or occupation. This is a real percentage increment in wage-and-salary earnings above the periodic, inflation-driven, cost-of-living adjustments that workers often receive. In the model this percentage may be constant to reflect a long-term, steady rise in productivity or may vary for different periods depending on the person’s age, experience, employment tenure, or other factors. In any event, the opportunity cost should be recorded starting the first year of pharmacy school.

#### 2.3.2. Benefits

The benefits derived from a pharmacy education investment stem from the professional wages and salaries earned as a pharmacist throughout the worklife span (i.e., the time between getting the first job as a pharmacist and retirement). For a practitioner with years of experience, historical earnings data may be used; if the earnings are forecasted for a prospective pharmacist, a realistic value should be chosen for the initial year and values for subsequent years should be projected into the future by adding an annual percentage increment in (real terms) productivity plus, if appropriate, the assumed annual inflation rate for the estimation of the nominal rate of return. The value of this percentage increment in productivity may or may not be equal to the percentage chosen for the opportunity cost depending on the assumptions that describe the specific person or group for whom the model is built.

It is virtually impossible to determine a uniform worklife stream of wages and salaries that fits all pharmacists. In markets characterized by a mobility of resources, wage-and-salary earnings are forged by the interplay of the supply of and demand for pharmacist services in a given location and at a given time. This is the level that pharmacists consider in making their marketwork-versus-leisure preference decisions and employers equate to their marginal revenue product in their quest for profit optimization subject to legal, institutional, and social constraints.

Wages and salaries may fluctuate widely due to different reasons. Gender is one of them. Nearly everywhere men and women play different roles dictated by society, whereby men are expected to bring home the primary source of income and women assume the primary household and childcare responsibilities. Compared to men, women often work fewer hours in the marketplace, are more likely to work part time, and interrupt their careers more frequently, all of which reduce their long-term income stream. Women also may be subject to institutional discrimination, which occurs when employers believe that hiring them instead of male pharmacists will lead to less job commitment and productivity; this practice inevitably results in lower wages and salaries as well as limited advancement opportunities [13,14,15,16].

Job-related preferences, in many instances conditioned by gender, also are important in projecting pharmacists’ long-term income streams. Pharmacists practice in different settings (i.e., retail, hospital, etc.), not all of which are equally remunerated. Regardless of practice setting, administrative positions generally draw higher pay than staff positions, although more often than not they are accompanied by greater responsibility and stress, which are not welcome by everyone [17,18,19,20,21]. Similarly, owning a pharmacy is more financially rewarding than working as an employee earning a wage or a salary [22]. Location of practice may be a factor, too; rural residence exerts a negative impact on pharmacists’ earnings [23,24] as well as the earnings of other healthcare professionals [25].

In addition, the effect of compensating differentials on earnings should be considered. These are job-related advantages and disadvantages for which pharmacists may be willing to trade off income. Included in this category are fringe benefits, commuting distance from home to worksite, advancement opportunities, scheduling flexibility, job atmosphere, and others that may enhance or depreciate a work position. Jobs with characteristics regarded by most workers as disadvantages normally pay more than jobs with advantages. In a mobile market, pharmacists choose among available jobs based on wages and salaries plus net advantages.

In summary, numerous factors affect using estimated income as an indicator of benefits derived from a pharmacy education investment, making its conceptualization a complex task. Furthermore, many of these factors are intangible and subjective in nature, and depend on tastes and preferences, lifestyles, and other choices that are unknown into the future or do not lend themselves readily to inter-personal comparisons. Yet, for purposes of calculating the rate of return to the investment, realistic wages and salaries must be projected over the pharmacist’s worklife span.

## 3. Results

The calculation of the rate of return to a pharmacy education investment, along the stipulations discussed here, would be simplified with a chart in which the estimated costs and benefits are summarized for each year of the investment span (see Table 1). There are eight headings in this table: year in which costs and benefits are recorded; cost of tuition, cost of school-related expenses other than tuition, financing cost, and opportunity cost, with the sum of all four costs yielding total cost; benefits, which consist of the income earned by the pharmacist; and the difference between benefits and costs. Year 1 is the first year of pharmacy school and Year *n* is the pharmacist’s expected retirement year; therefore, there are *n* annual data rows in this chart.

For purposes of combining estimated costs and benefits, a few significant years are identified in the table. Year *h* is the last year of pharmacy school and Year *h* + 1 is the year in which the individual begins working as a pharmacist. Thus, the pharmacist’s worklife span is *n − h* years. Year *k* is the year in which the pharmacist makes the last interest payment on the amount borrowed to finance his/her studies. As an illustration, consider an individual who undertakes a five-year pharmacy-school program at 21 years of age. He/she pays interest on student loans until ten years after graduation and eventually retires at 70 years of age. In this case, the expected retirement year is *n* = 50, the last year of pharmacy school is *h* = 5, the worklife span is *n* − *h* = 45, and the last interest payment on the amount borrowed to pay for pharmacy school occurs in Year *k* = 15.

The chart is applicable to the calculation of both the nominal and real rates of return; the difference would be whether or not costs and benefits are adjusted for inflation. For Year 1, T_1_ represents the cost of tuition, E_1_ represents the cost of the other school-related expenses, F_1_ represents the interest paid on the amount borrowed to pay for tuition and/or other school-related expenses, O_1_ represents the opportunity cost of not working, and C_1_ is the sum of these four cost categories. There is no B_1_ because the individual is not working as a pharmacist yet. In the following year, Year *2*, T_2_, E_2_, and F_2_ may or may not be the same as T_1_, E_1_, and F_1_, respectively, depending on the assumptions in the model; O_2_, however, would be equal to O_1_ plus the percentage gain in productivity (PG_2_) plus, if applicable, the anticipated annual inflation rate.

Beyond Year *h* there are no more costs of tuition and/or other school-related expenses, and the streams of benefits marked by the pharmacist’s income over the worklife span begins. The earnings for Year *h* + 1 may not reflect a full year’s pay if there is a gap between graduation and employment, so entering a fraction of B*_h_*_+ 1_ consistent with prorated work may be appropriate. Regardless of the existence of a gap, a realistic initial level of earnings, commensurate with the considerations discussed in Step 4, should be entered. This level also should take into account possible bonuses and the likelihood of additional pay because of overtime work. For the following year a percentage increment in productivity (PI*_h_*_+ 2_) should be added to the annualized amount of earnings of Year *h* + 1. This percentage increase has been denoted differently from the percentage added to the opportunity cost to accommodate the argument that a pharmacist’s percentage gain in productivity may be different from the gain in productivity of a non-pharmacist. Beyond Year *k* the opportunity cost is the only cost remaining in the model.

Once the chart is completed, the rate of return to a pharmacy education investment may be calculated as follows:

(1) $$\overset{n}{\underset{t=1}{\sum }}\frac{B_{t}-C_{t}}{{\left(1+r\right)}^{t}}=0$$

where B*_t_* is the amount of professional income earned by the pharmacist in Year *t*; C*_t_* is the total amount of costs incurred by the pharmacist in Year *t* (C*_t_* = T*_t_* + E*_t_* + F*_t_* + O*_t_*); and *r* is the rate of return; and where *t* = 1, …, *n*; and *n* is the number of years in the pharmacy education investment span, from entering pharmacy school to retirement.

The rate of return is the percentage per year that brings the stream of the annual difference between benefits and costs over the investment span down to zero. Other things equal, the higher the value of the rate of return is, the higher is the value of the denominator and the more profitable is the investment in a pharmacy education. The calculation of the values of B*_t_*, C*_t_*, and *n* is illustrated in Table 1. Specific applications to benefits, costs, and worklife span in the USA are presented by Carvajal and Popovici [6].

## 4. Discussion

The estimates obtained by applying the mechanisms outlined above are conditioned by a set of assumptions that are arbitrarily chosen to describe a specific individual or group of individuals. These assumptions cover, among other factors, the pharmacist’s worklife span, the costs incurred while in pharmacy school, and the income earned by working as a pharmacist. Intrinsic to all estimates is an element of uncertainty that must be incorporated into the process. Different assumptions may yield different estimates of costs, benefits, and the rate of return, so a procedure is needed to ascertain how sensitive the output (rate of return) is to a change in inputs (costs and benefits) induced by a change in the assumptions. This procedure is called sensitivity analysis.

Sensitivity analysis is a technique commonly used to test the robustness of results under conditions of uncertainty. It enables analysts to measure the responsiveness of the output to a change in one or more inputs. In this case, it recalculates the rate of return after altering one cost or benefit, which reflects a change in the assumptions, while holding everything else constant. For example, what might happen if the inflation rate after graduation from pharmacy school were higher than anticipated and the other assumptions remained the same? Aside from a greater disparity between the nominal and real rates of return, one would expect a drop in the real value of the interest paid (while part of the loan remained outstanding) on the amount borrowed to defray tuition and other school-related expenses; in other words, the real cost of financing, and consequently the total cost of acquiring a pharmacy education, would go down, which would increase the rate of return.

Other instances in which sensitivity analysis might be applied include the following: a decrease in the pharmacist’s worklife span, either due to a rise in the age at graduation or a drop in retirement age; an increase in the required length of the pharmacy school curriculum, prerequisites for admission, and/or mandatory internship service; an increase in tuition and/or school-related expenses; a rise in the interest rate charged to students who borrow money to pay for tuition and other expenses while attending pharmacy school; an increase in the opportunity cost of acquiring a pharmacy education due to the potential ability to earn a higher income level; a higher annual percentage increment in productivity applied to the opportunity cost; a decline in initial wages and salaries for pharmacists, brought about by an excess of practitioners in the labor market or a drop in the demand for pharmacist services; a lower annual percentage increment in productivity applied to income earned as a pharmacist; and a reduction in the number of hours worked, either voluntarily to accommodate non-work activities or because the available or desired jobs offer only part-time employment. Other things equal, all of these changes would reduce the rate of return to a pharmacy education either by increasing costs or diminishing income. By the same token, a change in the opposite direction in any of these assumptions would increase the rate of return.

The model should be sufficiently flexible to allow for practices such as bonding, which is not uncommon in some parts of the world, to affect inputs. A bonded pharmacy student is granted a scholarship by a government institution, say the Ministry of Health, that reduces or eliminates tuition fees in exchange for a commitment to work immediately after graduation, for a specified period, in the national health service, typically earning wages and salaries below market levels; if the student breaks this bond, he/she must pay back all tuition fees. In the model developed here, the bonded student would have zero or reduced tuition costs throughout pharmacy school, zero or reduced financing costs subsequently, and lower benefits (i.e., income) in the first few years after graduation, whereas a student who breaks the bond, or was unbonded, would experience both higher costs and benefits. The same adjustment would apply in countries where pharmacists are exempt from paying principal and/or interest on their student loans if they work in certain lower-paying or high-risk jobs that serve special populations.

Perhaps the most influential factors affecting adversely the rate of return to a pharmacy education are those that keep pharmacists from working and/or earning potentially higher levels of income. In an extreme case, individuals and/or groups who are not able to graduate from pharmacy school and work as pharmacists because of academic insufficiency, illness, death, administrative constraints, or any other reason lose their investment altogether (i.e., experience a negative rate of return). Less dramatically but more frequently, in most countries female practitioners experience lower rates of return compared to their male counterparts because they systematically earn lower wages and salaries and are promoted sparingly [26,27]. The same principle would apply to any other form of systematic income disparities, whether they occur because of ethnic group, national origin, political or religious affiliation, or any other basis. Any type of unequal treatment anywhere in the world inevitably reduces the affected individuals’ rates of return to their pharmacy education investment.

Sensitivity analysis is instrumental in ascertaining not only the direction, but also the magnitude of the output change due to a variation in an input. This is accomplished with the use of elasticity, sometimes called sensitivity, an indicator frequently used by economists and other social scientists. Elasticity measures the observed percentage change in the output (i.e., rate of return) divided by the percentage change in an input (i.e., cost or benefit) brought about by a varying assumption. For example, if the estimated rate of return increased by 6% (from 5.0% to 5.3%) due to an increase of 10% in the pharmacist’s anticipated income stream throughout the worklife span, the elasticity value would be 0.60.

Since elasticity is a ratio of two percentages, it is expressed in its own units. A positive (negative) elasticity sign denotes a direct (inverse) input–output relationship. If the absolute value of elasticity is less than one, the fraction indicates a less-than proportionate ratio or relatively weak response of the rate of return to a change in the cost or benefit of a pharmacy education, and the input effect on the rate of return is said to be inelastic; conversely, a value greater than one indicates a more-than proportionate ratio or stronger response of the rate of return to the cost or benefit change, and the effect is said to be elastic. Other things equal, the lower the calculated elasticity values are, the more robust the rate of return estimate is likely to be.

## 5. Limitations

In interpreting the estimates obtained by following the steps described in the Methods section, one must take into account several limitations inherent to the process. The accuracy of the results is based on many assumptions that over the long run may not be representative of a likely career pathway. The most obvious limitation is the inability of the model developed here to account for the multitude of administrative and regulatory variants found throughout the world, imposed by the social, economic, and political constraints of the healthcare and educational systems of each country. Applications to specific persons or groups may require additions, deletions, and/or changes not identified explicitly in this article. Hopefully the structural framework possesses the flexibility to accommodate these variants and still yield meaningful estimates.

A second limitation is that the benefits and costs identified in the methodology are independent of taxes. A more progressive income tax structure (i.e., the tax rate goes up as the amount of taxable income increases) may affect adversely the pharmacist’s willingness to work, thereby lowering his/her potential income stream and, other things equal, also reducing the rate of return to his/her pharmacy education investment. Factors such as income from non-labor sources, wages and salaries of other family members, and other earnings that contribute to taxable household income are not considered in the model. Along the same lines, interest payments from the education loan may be tax deductible, in which case estimated total costs would decline and the rate of return would increase.

Neither does the model consider disparities in purchasing power in different locations, which is a third limitation of the study. For example, consider two pharmacists under identical conditions, except that one lives in an area where the cost of living is relatively high and the other pharmacist lives in another area of the same country where the cost of living is lower. The tenets of purchasing power parity suggest that the first pharmacist be paid higher wages and salaries to compensate for the higher cost of living, so both of them would be able to purchase the same goods and services and enjoy a comparable standard of living. The pharmacist being paid a higher nominal income, however, would accrue an artificially greater long-term stream of benefits, and consequently a higher rate of return, than the other pharmacist, which would portray a distorted picture of the relationship between both of them.

Another limitation is the oftentimes uncertain nature of the premises and assumptions that govern the calculations. When a candidate gains admission into pharmacy school, it is difficult to ascertain whether he/she will eventually practice in a retail, hospital, or other venue; reduce the number of hours worked or drop out of the labor force altogether for a significant period due to childcaring or other non-market responsibilities; or experience an unexpected event. Recently developing technologies and changes in the practice of the profession impact strongly on any estimates. Other macrofactors such as financial crises, pandemics, and wars, as well as microfactors such as personal and family illness, unforeseen opportunities, and choices of income versus lifestyle also influence pharmacists’ decisions and wellbeing. Yet, these considerations affect the rate of return and choices must be made in the model in order to project the long-term streams of expenses and income that determine costs and benefits. Moreover there are no objective criteria to identify which assumptions are “better” or “more realistic” in the estimation of costs and benefits; each assumption reflects circumstances and perceptions that may change over time (i.e., from student pharmacist to practicing pharmacist with 20 years of experience), and the “best” choice may be one that optimizes job, career, or even life satisfaction at the expense of the financial option.

Still another limitation is the inability of the model to answer whether or not the financial benefits received from, and ultimately the rate of return to, studying pharmacy make the investment in a pharmacy education the best possible choice for a person or group. The estimated rate of return may answer whether or not pharmacy is financially viable for a specific individual or group of individuals in a given place and at a given time; however, while the anticipated rate of return is an important consideration, other factors such as aptitude, motivation, and professional self-projected imaging are important as well. Over the long run in the worklife span, job satisfaction frequently becomes more important to pharmacists than the income they earn or other financial considerations [28].

## 6. Conclusions

The portrayal in this article of pharmacy education as a human capital investment, conditioned by long-term expenses and income streams that may vary not only across countries but also within national boundaries and through time, may fall out of the comfort zone for practitioners geared primarily toward the world of drug discovery, development, and utilization. Commonly friendly concepts such as patient care, molecular formulae, and drug interaction have been displaced by investment costs, benefits, and other economic pursuits that focus on the sustainability of pharmacy as a profession, reducing all that seems relevant to currency units and largely ignoring the intangible zeal that drives many practitioners in their pharmacy school years and subsequently through their professional lives. Critics may argue that although the model developed here may be accurate in hindsight, it lacks the ability to be predictive over the pharmacist’s worklife span. Nonetheless this approach is necessary to establish the financial viability of pharmacy and compare it to the viability of other professions as well as the viability of pharmacy among various locations.

## Figures and Tables

**Table 1:** Hypothetical chart summarizing estimated annual costs and benefits related to a pharmacy education investment.

Year (*t*)	Costs *					Benefits (B*_t_*)	Benefits Minus Costs (B*_t_* − C*_t_*)
	School-Related		Financing (F*_t_*)	Opportunity (O*_t_*)	Total (C*_t_*)		
	Tuition (T*_t_*)	Expenses (E*_t_*)					
1	T_1_	E_1_	F_1_	O_1_	C_1_	-	−C_1_
2	T_2_	E_2_	F_2_	O_1_ + PG_2_	C_2_	-	−C_2_
…							
*h*	T*_h_*	E*_h_*	F*_h_*	O*_h_*_− 1_ + PG*_h_*	C*_h_*	-	−C*_h_*
*h* + 1	-	-	F*_h_*_+ 1_	O*_h_* + PG*_h_*_+ 1_	C*_h_*_+ 1_	B*_h_*_+ 1_	B*_h_*_+ 1_ − C*_h_*_+ 1_
*h* + 2	-	-	F*_h_*_+ 2_	O*_h_*_+ 1_ + PG*_h_*_+ 2_	C*_h_*_+ 2_	B*_h_*_+ 1_ + PI*_h_*_+ 2_	B*_h_*_+ 2_ − C*_h_*_+ 2_
…							
*k*	-	-	F*_k_*	O*_k_*_− 1_ + PG*_k_*	C*_k_*	B*_k_*_− 1_ + PI*_k_*	B*_k_* − C*_k_*
*k* + 1	-	-	-	O*_k_* + PG*_k +_*_1_	C*_k +_*_1_	B*_k_* + PI*_k_*_+ 1_	B*_k_*_+ 1_ − C*_k_*_+ 1_
…							
*n*	-	-	-	O*_n_*_− 1_ + PG*_n_*	C*_n_*	B*_n_*_− 1_ + PI*_n_*	B*_n_* − C*_n_*

* C*_t_* = T*_t_* + E*_t_* + F*_t_* + O*_t_*.
